# Shredded Bacterial Cellulose as a Potential Carrier of Polyphenols Derived from Apple Waste Applying Rapid Solid–Liquid Dynamic Extraction

**DOI:** 10.1021/acsomega.4c10689

**Published:** 2025-04-03

**Authors:** Roberta Baldassini, Brunella Corrado, Elena Lagreca, Raffaele Vecchione, Alma Sardo, Daniele Naviglio, Paolo Antonio Netti

**Affiliations:** † Knowledge for Business, Via Manzoni 110, 80123 Naples, Italy; ‡ Interdisciplinary Research Centre on Biomaterials (CRIB), University of Naples Federico II, Piazzale Tecchio 80, 80125 Naples, Italy; § Istituto Italiano di Tecnologia, Largo Barsanti e Matteucci 53, 80125 Naples, Italy; ∥ Department of Chemical, Materials and Industrial Production Engineering, University of Naples Federico II, Piazzale Tecchio 80, 80125 Naples, Italy; ⊥ Department of Veterinary Medicine and Animal Production, University of Naples Federico II, Via Federico Delpino, 80137 Naples, Italy; # Department of Chemical Sciences, University of Naples Federico II, Via Cintia 4, 80126 Naples, Italy

## Abstract

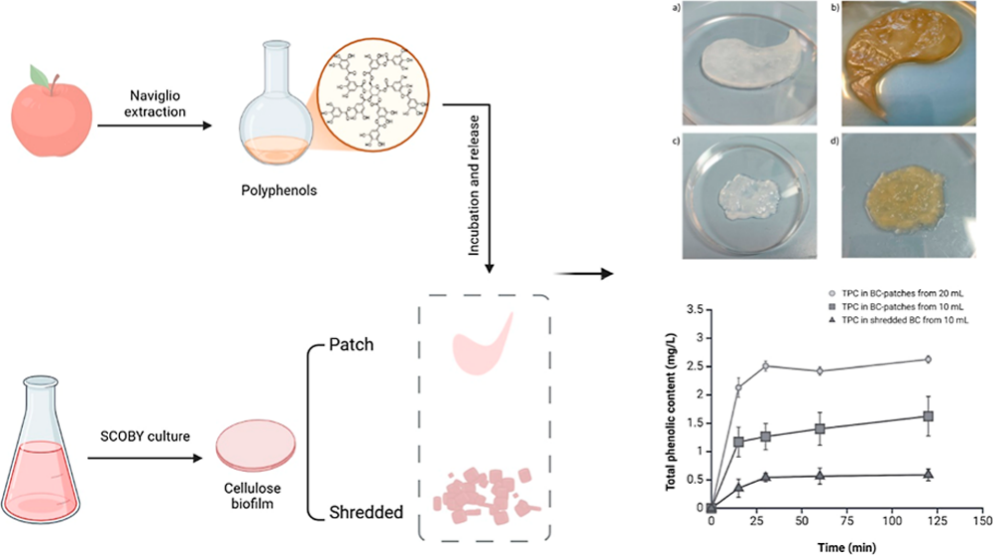

The production and application of bacterial cellulose (BC) from nonpathogenic microorganisms, such as *Acetobacter* species, have gained popularity due to its high-water absorption capacity and mechanical strength. Although BC is already used as an additive to modulate the texture of some cosmetic and food formulations, it has not yet been characterized as a carrier of bioactive compounds in such formulations. This study explores the production and characterization of shredded-BC incorporating polyphenolic compounds extracted from waste apples using rapid solid–liquid dynamic extraction (RSLDE) at room temperature. While shredding did not alter BC’s inner microstructure, the increased surface-to-volume ratio and simplified tridimensional structure significantly enhanced the absorption and release kinetics of polyphenols, enabling faster delivery. These findings highlight the potential of shredded-BC as a particle-based ingredient for cosmetic masks, facilitating the rapid release of antioxidant compounds and offering new opportunities for the development of BC cosmetic formulations capable of controlling the release rate of bioactive compounds by modulating their size.

## 1 Introduction

Cellulose is a biocompatible, renewable, nontoxic, and biodegradable biopolymer that is present in nature. Together with plant-derived cellulose, this material can also be synthesized by several acetic acid-producing bacteria such as *Acetobacter*, *Gluconobacter*, *Gluconacetobacter* and *Komagataeibacter*, commonly found in fermented foods such as vinegar, kombucha, nata de coco and rotting fruit. These bacteria can oxidize alcohols, aldehydes, and sugars in the presence of oxygen into acetic acid, producing a moist extracellular matrix made of crystalline cellulose as protection against desiccation and UV damage.^1^ In this form, cellulose is known as biocellulose or bacterial cellulose (BC). Although it possesses the same molecular structure as plant cellulose, BC is free of lignin, pectin, hemicellulose, and other natural organic products. Additionally, BC has unique advantages compared with plant cellulose, such as ultrafine network structure, from elementary fibrils to larger units like microfibrils and bundles finally packed into fibers, ultimately contributing to high water absorption capacity, mechanical strength, and high crystallinity.^2,3^

All these characteristics make BC a material suitable for many applications, including cosmetics,^1^ with a growing trend in utilizing BC in cosmetic masks due to their effectiveness, ease of application and low costs.^4^

In literature, numerous studies focus on the use of cellulose layers as carriers for antioxidant substances in the formulation of antiaging, lifting, purifying and regenerative masks^5^ with plenty of compounds that can be coupled to cellulose, both hydrophilic, such as caffeine,^6^ oat, rosemary, calendula and propolis extracts,^7^ and lipophilic, such as Coenzyme Q10.^8^ Among plant extracts, polyphenolic compounds are potent antioxidants.^9−11^ They can bind to cellulose with a binding capacity strongly influenced by the degree of aggregation of the cellulose microfibrils and by the availability of hydroxyl groups in the cellulose structure with consequent van der Waals forces and hydrogen bonds. Therefore, the greater the number of hydroxyl groups and aromatic rings, the greater the possible interactions.^12−14^

In particular, there are studies combining BC with various plant extracts, such as in Nowak et al. study where *Epilobium angustifolium* L. extract is used for topical administration of antioxidant phenols to the skin.^15^ Fatima et al. on the other hand, added antimicrobial activity to BC through an extract of *Euclea schimperi*.^16^ Another example was studied by Indrianingsih et al. where Green tea leaf extract (*Camellia sinensis*), rosella flower petals (*Hibiscus sabdariffa*) and *Hibiscus rosa-sinensis L.* flower extract were explored for their antioxidant and antibacterial properties.^17^ Although apple polyphenols are a versatile and highly effective option for formulating antiaging, protective, and regenerative cosmetic products, offering the added advantage of being gentle and well-tolerated by skin, they have yet to be explored in combination with BC. There are only a few examples of apple juice utilized as a culture medium for BC.^18^ Additionally, even though skin masks can be formulated in different forms such as gel, cream, sheet, or paste, each offering specific benefits, most cosmetic applications involve BC in sheet form, and exhaustive research on shredded-BC has yet to be conducted.

Although, there are studies on micronized BC as additive agents for food and cosmetic products^19,20^ to the best of our knowledge no literature reports on shredded-BC as a carrier of bioactive compounds, and consequently no data concerning the uptake and release from shredded-BC is available. So, for the first time, in this study, we used shredded-BC as carrier and compared it with BC layer in terms of absorption and release, in order to assess its advantages in absorption and release rates. In this way, it was possible to highlight the potential of shredded-BC as a carrier for the complete and rapid release of the loaded plant extract, guaranteeing its active form. Furthermore, through these incubation and release studies, it emerged the dependence on the BC size opening new avenues for the development of BC cosmetic formulations capable of controlling the release rate of bioactive compounds by modulating their size.

Our experimental methodology involved the cultivation of BC using a symbiotic culture of bacteria and yeast (SCOBY) and the subsequent preparation of shredded-BC samples. An initial characterization of the two types of BC was made using scanning electron microscopy (SEM) to point out any similarities or differences between the two BC samples. This analysis examined BC’s structural, morphological, and functional properties, crucial for understanding its potential as a versatile biomaterial for various biotechnological applications.

Meanwhile, a polyphenol extract was prepared using Italian apples and an RSLDE technique. Then, a comprehensive characterization of the apple polyphenol extract (APE) through various analytical methods, including the Folin-Ciocalteu (FC) assay and the 2,2-diphenyl-1-picrylhydrazyl (DPPH) assay, was carried out.

Once the initial materials were obtained, APE incubation and release tests were conducted, followed by kinetics postanalysis through FC assay, Fourier-transform infrared spectroscopy (FT-IR), and biocompatibility assessments.

The results demonstrated the potential of shredded-BC as an effective carrier material. Overall, this study contributes to the growing body of knowledge concerning the utilization of BC as a platform for controlled release systems and sheds light on the potential benefits of shredded-BC as an alternative to conventional cellulose forms, paving the way for further exploration and development of innovative biomaterial-based solutions for diverse applications in healthcare, pharmaceuticals, and related fields. In this way, the use of shredded-BC in this unconventional shape, instead of typical layered structure, could further highlight its capacity and versatility as a carrier material.

## 2 Experimental Section

### 2.1 Materials

Apple waste of Annurca variety production was sampled in Campania, Italy, from a local agricultural company and provided by Tec up Startup Laboratories. Apples wastes were stored at −20 °C until use.

Dulbecco’s Phosphate Buffered Saline 1× (Microgem S.R.L., United States) was used for polyphenol release studies. Human dermal fibroblasts (HDFs) were purchased from the American Type Culture Collection (ATCC, United States). All other reagents and solvents were of analytical grade: Folin-Ciocalteu reagent, ethanol 99.8% v/v, DPPH, and methanol were purchased from Sigma-Aldrich (Germany), and sodium carbonate anhydrous from Carlo Erba (France).

### 2.2 Methods

#### 2.2.1 Media Preparation and SCOBY Culture

For the culture of Kombucha SCOBY (KEFIRA), a tea broth was prepared according to a previously described protocol.^8^ Briefly, 860 mL of deionized water (dH_2_O) was boiled before adding 140 g/L of glucose; 20 g of black tea bags were added and steeped for 10 min. Consequently, the tea bags were removed, the sweetened tea was cooled at room temperature; then, apple vinegar (140 mL/L) was added. The medium was autoclaved at 121 °C for 15 min. One piece (1 × 1 cm) of SCOBY was aseptically added into the liquid broth (20 mL within a 50 mL volume tube) and cultured in static conditions for 3 days, acting as a starter. For BC layer production, starters of the SCOBY were cultured in 50 mL tubes in controlled fermentation conditions. The fermentation occurred in a dark CO_2_ incubator (Stuart SI60D, Thermo Fisher, United States) in a controlled humidified atmosphere (≥80%) with a constant temperature of 30 °C for 3 days, guaranteeing an optimal environment for symbiont growth. The cap of the tube was removed, and perforated parafilm, previously sterilized, was used to cover the lid and increase the exchange of O_2_ with the external surface. This process was repeated in triplicates. BC layers were collected after 3 days of culture and autoclaved. Bleaching was carried out with 3% v/v hydrogen peroxide, and then, the layers were rinsed with distilled water to neutrality. Lastly, to shape cellulose layers in patch form, some were cut with a stamp and collected for further analysis.

#### 2.2.2 Shredded-BC Production

BC layers described in the previous section were roughly cut into small pieces and mixed with an equal weight of distilled water. Then they were shredded using a household electric blender for 5 min and subsequently collected.

#### 2.2.3 Ultrastructural Characterization of the BC Layer and Shredded-BC

To perform ultrastructural analysis of the fibrillar structure of BC, BC layers and shredded-BC were primarily fixed with 4% (w/v) paraformaldehyde. Then, they were fixed with 2.5% (w/v) of glutaraldehyde in 0.1 M of sodium cacodylate and were left for 1 h at room temperature. After this time, samples were washed thrice for 10 min in 0.1 M sodium cacodylate and buffered at room temperature. They were then buffered with 1% (w/v) osmium tetroxide (OsO_4_) in 0.1 M sodium cacodylate for 1 h at 4 °C and, afterward again, washed thrice with 0.1 M sucrose buffer solution. Dehydration was performed on the samples using ethanol at 30, 50, 70, and 95% hydroalcoholic solutions for 30 min at 4 °C. Finally, three times, 100% ethanol was applied for 30 min at room temperature. After a complete dehydration of the samples performed by a critical point dryer (CPD, Emitech K850, United Kingdom), they were gold-coated and then acquired by scanning electron microscopy (SEM) (Zeiss, S400, Germany). SEM images (1024 × 768 pixels) were obtained and then analyzed using the ImageJ DiameterJ plugin. More specifically, the images were first segmented by algorithms provided by “DiameterJ Segment” to convert the image into binary forms. Then, segmented images were processed by DiameterJ to measure cellulose network parameters, such as the mean pore area and porosity percentage.

#### 2.2.4 Extraction of Polyphenols through Naviglio Extractor

**Figure 1:**
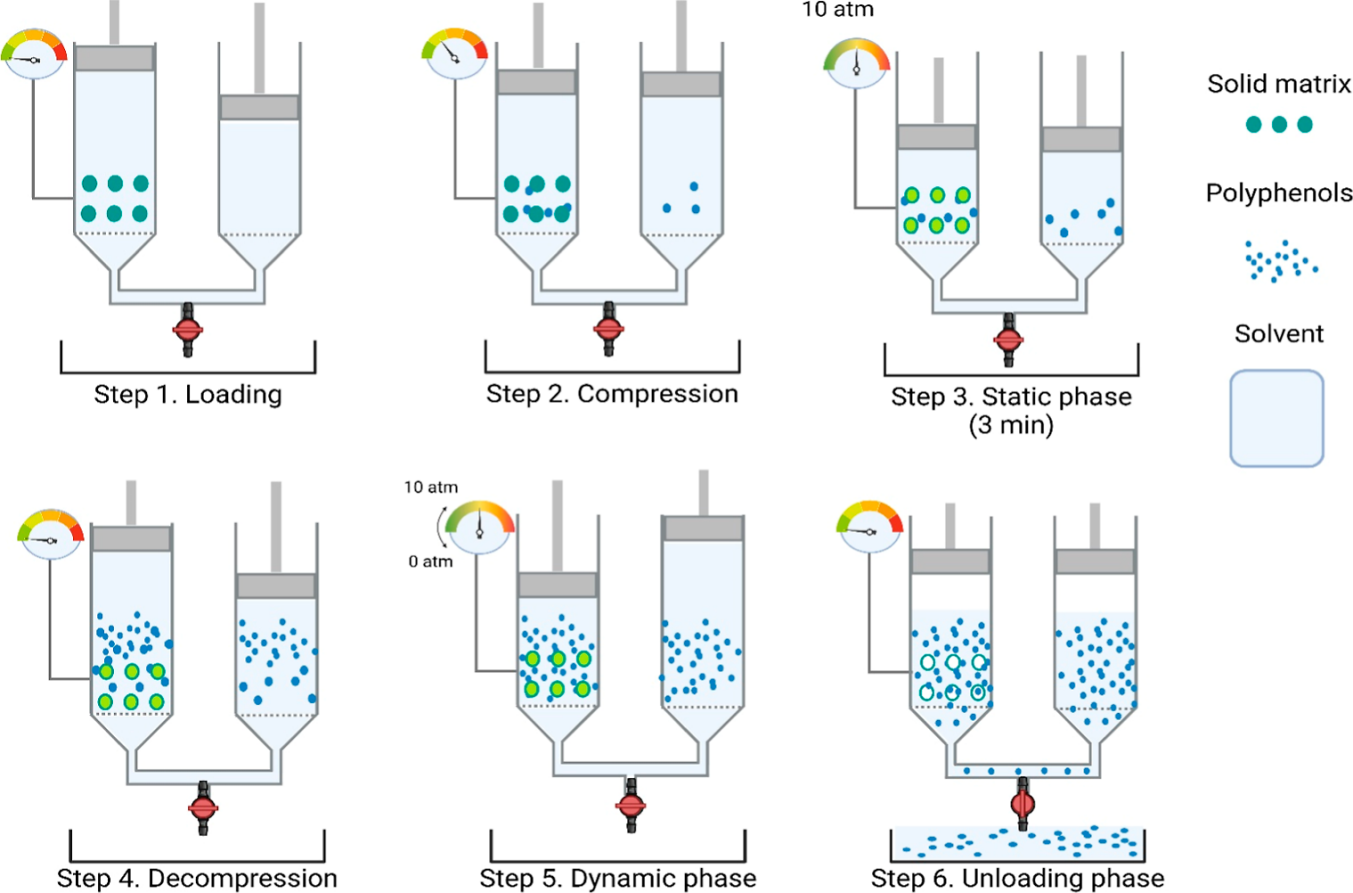
Schematic representation of the Naviglio extractor process for extracting polyphenols from apple waste using RSLDE.

Considering the easy thermal degradation of polyphenols at high temperatures, we selected rapid solid–liquid extraction (RSLDE) as extraction technology since it has already proven reliable for polyphenols.^21^ The advantage of RSLDE is that it does not require heating the extraction system since the action is mechanical. Polyphenols were extracted using Naviglio Extractor (500 mL model, Atlas Filtri Engineering, Italy). Figure 1 shows the setup and the process that takes place within the extractor’s chamber.

Briefly, the extractor works by alternating a static and a dynamic phase; during the first phase (static), the extraction solvent is brought to a pressure of about 10 atm and is maintained in this state for a sufficient time to let the solvent penetrate and reach an equilibrium between the internal and external pressure of the solid matrix (generally 3 min); at the end of the static phase, a dynamic phase begins, which generates a rapid drop of liquid under pressure to atmospheric pressure. The negative pressure gradient between the inner and outlet of the solid matrix causes a fast flow of liquid from the inside to the outside of the matrix and enables extraction.^22^

Approximately 250 gr of seedless and juice-free apples were weighed and premacerated with a hydroalcoholic solution of 400 mL ethanol 99.8% (v/v). After 10 min, apples and ethanol were transferred into the extraction chamber of Naviglio extractor. The APE was obtained with the following conditions: 3 min static phase followed by 10 strokes dynamic phase for a total of 576 cycles (48 h). Extraction took place at room temperature and under a pressure of approximately 10 atm. According to a previously reported protocol,^23^ 15 mL samples were collected at 2, 4, 24 and 48 h intervals after being appropriately filtered with absorbent paper. After each sample collection, an equal volume of hydroalcoholic solution was added to the extraction chamber to avoid pressure decrease. The filtered extracts were stored at room temperature in the dark.

#### 2.2.5 Characterization of the Extract

##### 2.2.5.1 Analysis of total Phenolic Content by Folin–Ciocalteu Reagent

FC reagent was used for the colorimetric determination of phenols and polyphenols.^24^ Briefly, 100 μL of the sample was collected, and 500 μL of FC reagent was added. Then, 1.5 mL of 20% sodium carbonate was added and made up to volume with distilled water in a 10 mL volumetric flask. The solution was mixed for 1 min and then kept in the dark. After 1 h, a spectrophotometric measure was made at a wavelength of 765 nm (Bausch & Lomb, SPECTRONIC 21). Standard gallic acid (GA) was used to obtain the calibration curve, and the results for TPC content were expressed as mg of gallic acid equivalents (GAE) per 1 L ± SD of solvent.

##### 2.2.5.2 DPPH Assay

For DPPH assay,^25^ a 0.005% (w/v) solution of DPPH in methanol was freshly prepared. Then, 50 μL of the sample was added to 900 μL of the reagent solution, and the reaction was let to happen. After 1 h in the dark, the absorbance was measured spectrophotometrically at a wavelength of 517 nm using a Bausch & Lomb SPECTRONIC 21.

#### 2.2.6 Incubation and Release

For incubation, 3 g of BC layers were immersed in 10 and 20 mL of 48 h APE, respectively (referred as patch-10 and patch-20), at room temperature and under gentle stirring at 120 rpm on an orbital shaker (Orbital Shaker Incubator ES-20, BioSan, Latvia). According to previous studies,^14^ the binding of polyphenols to the cellulose matrix increases during the first 60 min, reaching a plateau after 120 min of incubation. Samples were collected at 15, 30, 60 and 120 min for further analysis. Incubation efficiency was calculated by subtracting the amount of polyphenols remaining in the collected sample solutions from the initial amount in the APE, as determined by the FC assay.

The previously incubated patch-10 was suspended in 10 mL of PBS at 37 °C on the same orbital shaker to study polyphenols release. Assuming potential local use of BC layers, samples were collected at increasing time intervals of 5, 10, 15, 20, and 30 min. Release efficiency was obtained again through the FC assay, considering the amount of polyphenols absorbed by the corresponding layer during incubation.

Regarding shredded-BC (referred as shredded-10), the incubation and release experiments were conducted under the same conditions. In particular, 3 g of shredded-BC were incubated in 10 mL of 48 h APE for a total of 120 min of incubation. For the release study shredded-10 was suspended in 10 mL of PBS for a total of 30 min. The collected samples were centrifuged (MicroCL 21R, Thermo Fisher, Germany) at 14.000 rpm for 5 min to remove cellulose sediments and the supernatant was recovered for analysis. All experiments were performed in triplicate to allow for statistical analysis.

#### 2.2.7 Fourier-Transform Infrared Spectroscopy

As outlined in a previous study,^8^ the chemical compositions of the BC layer, shredded-BC and APE were examined using Fourier-transform infrared spectroscopy (FT-IR). The assessments were conducted within the 500–4000 cm^–1^ range, employing absorption or transmission modes with specific parameters (64 scans, 4 cm^–1^ resolution) (Thermo Fisher Scientific Instruments, Nicolet 6700, United States). Before analysis, the samples underwent a 24 h oven drying process to facilitate water evaporation. Subsequently, the acquired spectra underwent attenuated total reflection (ATR) correction, smoothing, and baseline correction to achieve normalization with OMINC Spectra software.

#### 2.2.8 Biocompatibility Tests

##### 2.2.8.1 Cell Culture

Human dermal fibroblasts (HDFs) were used to assess the biocompatibility of the BC layers. The HDF cells were cultured in 150 cm^2^ polystyrene tissue culture flasks (Corning Inc., United States) in enriched minimum essential medium (MEM) Eagle composed of MEM (Sigma-Aldrich, Italy), 20% (w/v) of fetal bovine serum (FBS) (Sigma-Aldrich, Italy), 2% of nonessential amino acids (EuroClone ECB3054D, Italy), 1% of l-glutamine (Lonza 17-605E, Switzerland), and 1% of penicillin/streptomycin (Sigma-Aldrich, Italy). The medium was changed every 2 days until reaching 90% confluence.

##### 2.2.8.2 Viability Assay

The effect of the BC layers on HDF cells viability was assessed using a direct contact method. Before analysis, BC layers were sterilized under UV light for 1 h, punched with an 11 mm puncher and put in a 48-well plate. Fibronectin solution (50 μg/mL) was used to functionalize BC layers before cell seeding. HDFs were harvested at 90% confluence and then detached by washing three times with PBS and incubated with trypsin–ethylenediaminetetraacetic acid (0.25% (*w*/*v*) trypsin, 1 mM EDTA; Microtech, Italy) for 5 min at 37 °C. HDF cells were seeded on the BC layers at 1 × 10^5^ cell density per BC layer. According to the manufacturer’s protocol, the cell viability was determined using the Alamar Blue assay (Thermo Fisher, United States) at 24, 48, and 72 h. Briefly, cells were incubated with 10% v/v Alamar Blue for 4 h, after which 100 μL of the supernatant from the culture medium was moved to a 96-well plate in triplicates, and the absorbance was measured using a plate reader (Microplate Readers PerkinElmer, Austria) at the wavelengths 570 and 600 nm. HDF monolayer in a 48-well plate was used as a control.

#### 2.2.9 Statistical Analysis

Data are presented as mean values and standard deviation. Statistical significance between sample populations is evaluated by using ANOVA and Tukey’s post hoc test at the significance level of *p* < 0.01. All the experiments are conducted in triplicate.

## 3 Results and Discussion

**Figure 2:**
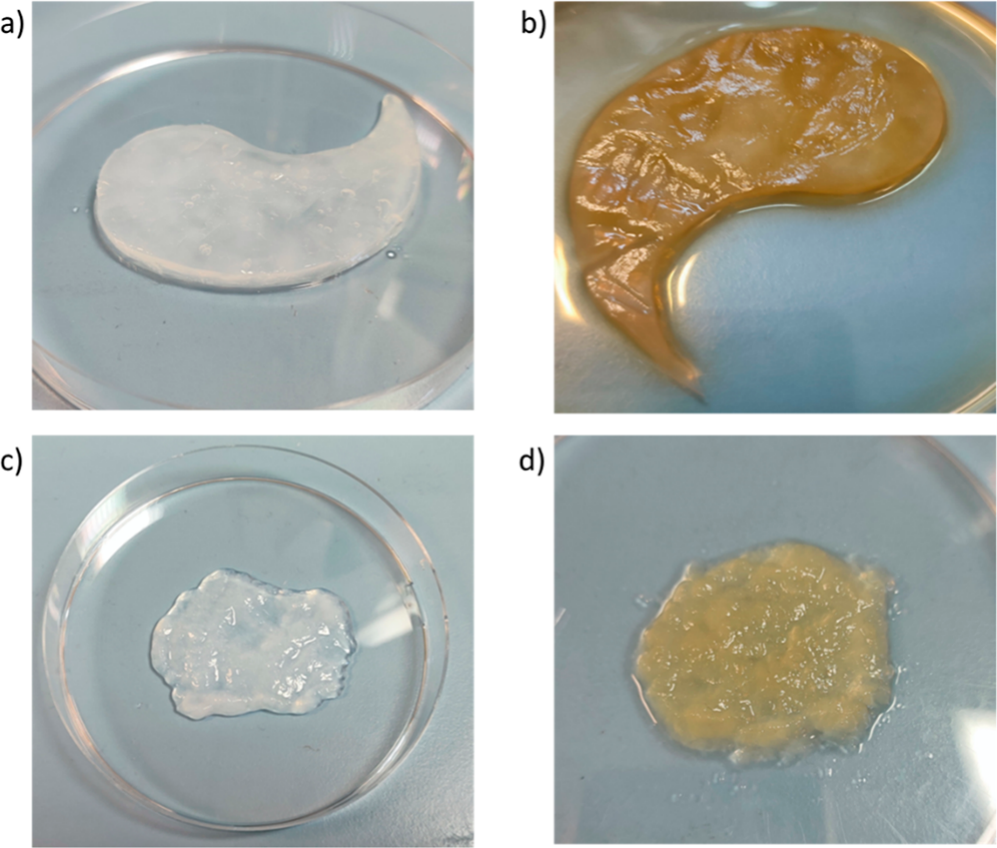
Images of BC patch and shredded-BC before (a,c) and after incubation with APE (b,d).

We analyzed the structural, morphological, and functional properties, as well as the absorption and release amounts and rates, to compare the BC layer and shredded-BC. The purpose was to evaluate the potential advantages of shredded-BC as a versatile carrier for loading plant extracts entirely and rapidly. Figure 2 shows the appearance of BC patch and shredded-BC samples before (a,c) and after incubation (b,d) with APE.

### 3.1 Ultrastructural Characterization of the BC Layer and Shredded-BC

**Figure 3:**
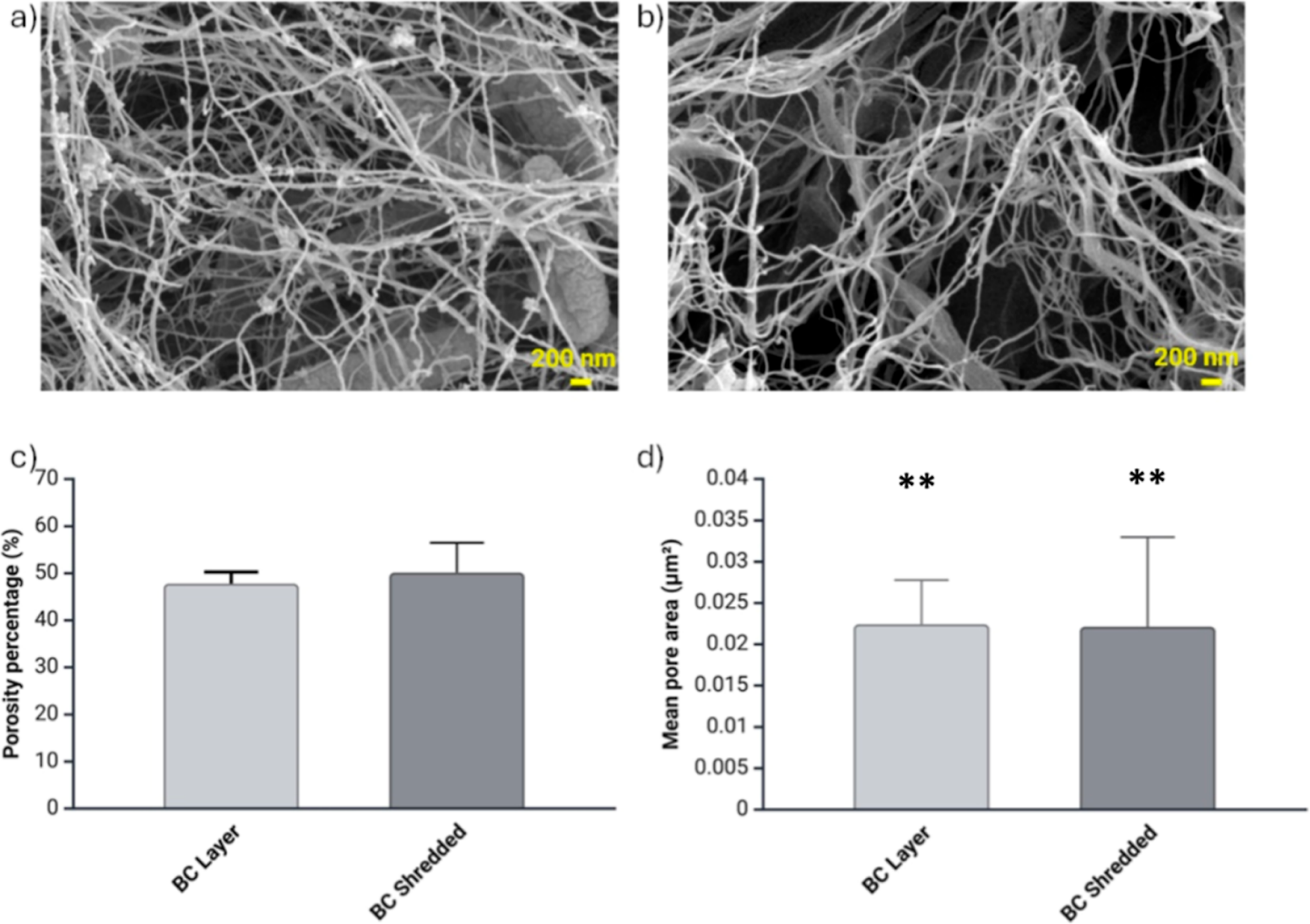
SEM images of the BC layer (a) and shredded-BC samples (b) scale bar 200 nm. By using the diameterJ plug (ImageJ), porosity percentage (c) and mean pore area (d) were measured for both the BC layer and shredded-BC samples. Data is expressed by mean and standard deviation. Statistical significance is assessed through the Anova test (***p < 0.005, **p < 0.01, *p < 0.05, not significant when not shown).

BC layer and shredded samples were processed for ultrastructural characterization. SEM images are shown in Figure 3 and provide visual confirmation of the cellulose network within both the BC layer (Figure 3a) and shredded-BC (Figure 3b) samples. A detailed analysis was performed to characterize the ultrastructural aspects further focusing on the percentage of porosity (Figure 3c) and mean pore area (Figure 3d). As expected, the ultrastructural results are in line with the porosity percentage and mean pore area found in the literature.^26^ Moreover, comparing the BC layer and shredded-BC samples did not reveal substantial differences. In specific quantitative terms, the mean pore area for the BC layer was 0.0224 ± 0.0053 and 0.0220 ± 0.0032 μm^2^ for shredded-BC. For the BC layer, the percentage of porosity was determined to be 47.8 ± 0.02%, while the shredded-BC samples exhibited a slightly higher porosity rate at 50.2 ± 0.06%.

This similarity in pore area and percentage of porosity suggests a consistent and comparable structural pattern in both the intact BC layer and the shredded-BC samples, emphasizing the robust nature of the cellulose network in maintaining its characteristic features even after shredding.

### 3.2 Characterization of Apple Polyphenol Extract

To characterize the APE obtained from RSLDE, 15 mL samples were collected at various time points, and the TPC was determined using the FC assay. The absorption signal showed a maximum of 765 nm, the analytical wavelength for measuring the TPC.

**Figure 4:**
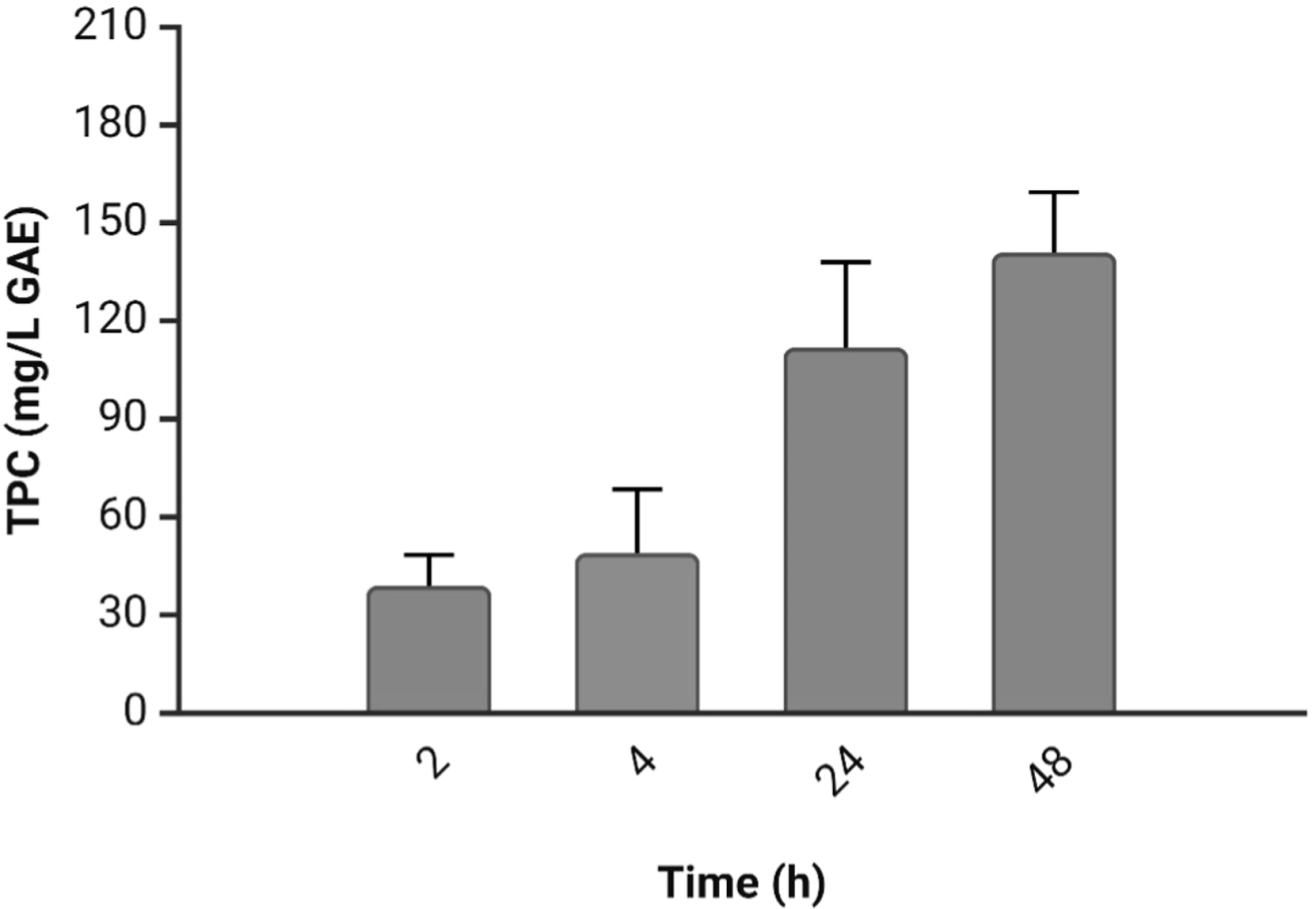
Amount of polyphenols extracted from 100 g of apples in 1 L solution at different incubation times (2, 4, 24, and 48 h), expressed as mg/L gallic acid equivalents (GAE). Data are shown as mean ± standard deviation (SD).

The experiment was performed in triplicate, and the cumulative results were processed to express the content as mg of gallic acid equivalents (GAE) per 1 L ± SD of the solution, starting from 100 g of fresh weight (FW) of the initial matrix. As shown in Figure 4 and consistent with the literature,^27^ the extraction process yielded approximately 140.90 mg/L of polyphenols from 100 g of plant matrix over 48 h, with a notable concentration increase over 24 h. The extraction process was stopped after 48 h to maximize polyphenol recovery while avoiding unnecessarily prolonged extraction times.

The DPPH assay was conducted on the same samples collected at 2, 4, 24, and 48 h to assess the radical scavenging capacity of the extracted polyphenol mixture. For this assay, the analyzed extract volume was reduced from 100 to 50 μL due to the complete suppression of the DPPH peak after 24 h, which resulted in a radical scavenging capacity close to 100%.

**Figure 5:**
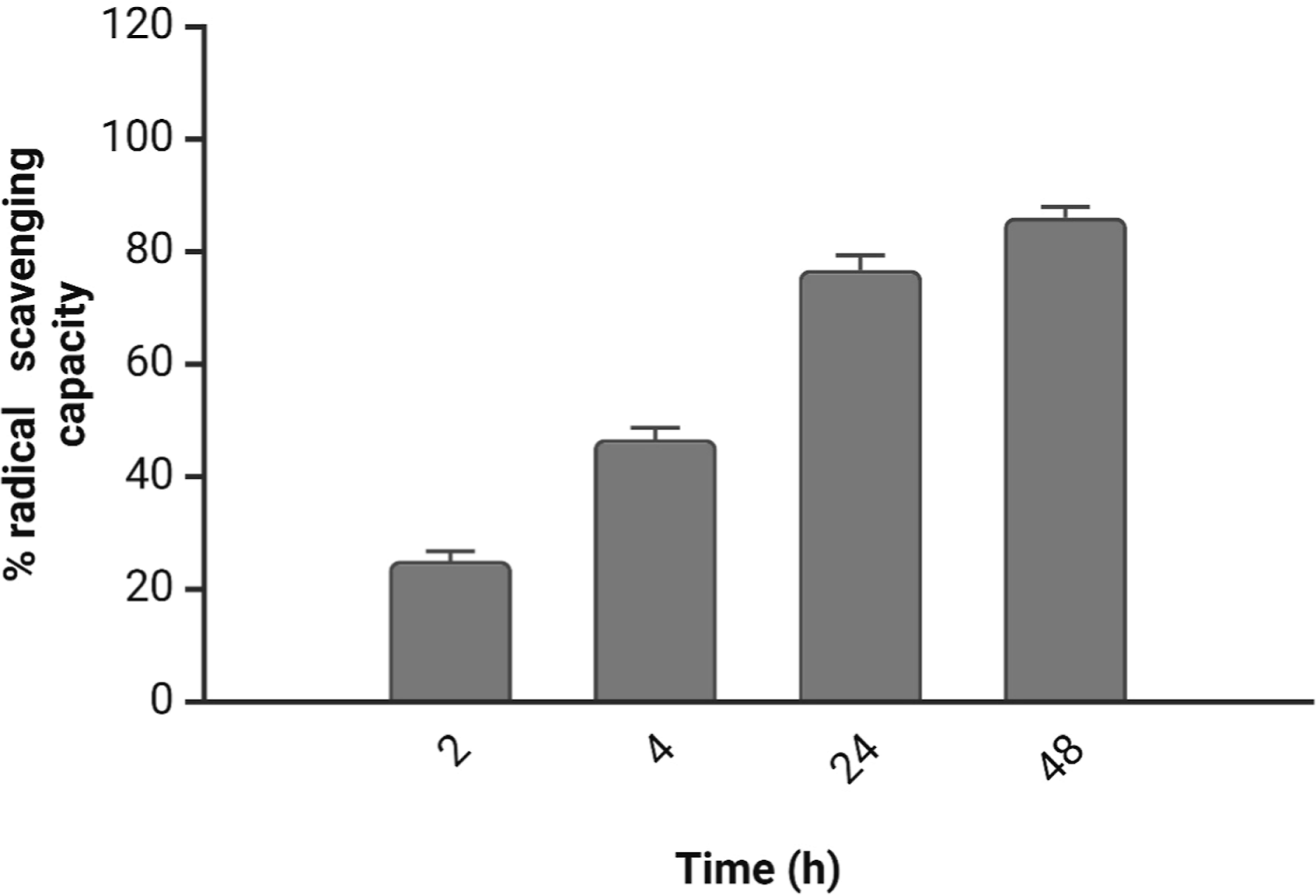
Radical scavenging capacity (%) of the polyphenol extract from 100 g of apples in 1 L solution over a 48 h incubation period. Data are expressed as mean ± standard deviation.

The cumulative results are shown in Figure 5, demonstrating how the increased concentration of polyphenols correlates with a rise in the radical scavenging capacity of the extract.

In addition, the alcoholic degree of the APE was measured using an alcohol meter upon completion of the extraction process. Despite using 99.8% (v/v) ethanol as the solvent, the high-water content of the initial matrix reduced the alcohol concentration, resulting in a final hydroalcoholic solution at 67% (v/v).

Lastly, before starting incubation experiments, the chemical stability of the APE over time was evaluated. The FC and DPPH assays were repeated 6 months later, yielding TPC and radical scavenging capacity values comparable to the initial measurements. This indicates high stability of the extract when stored in the dark (data not shown).

### 3.3 Absorption Comparison between BC Patches and Shredded-BC

**Figure 6:**
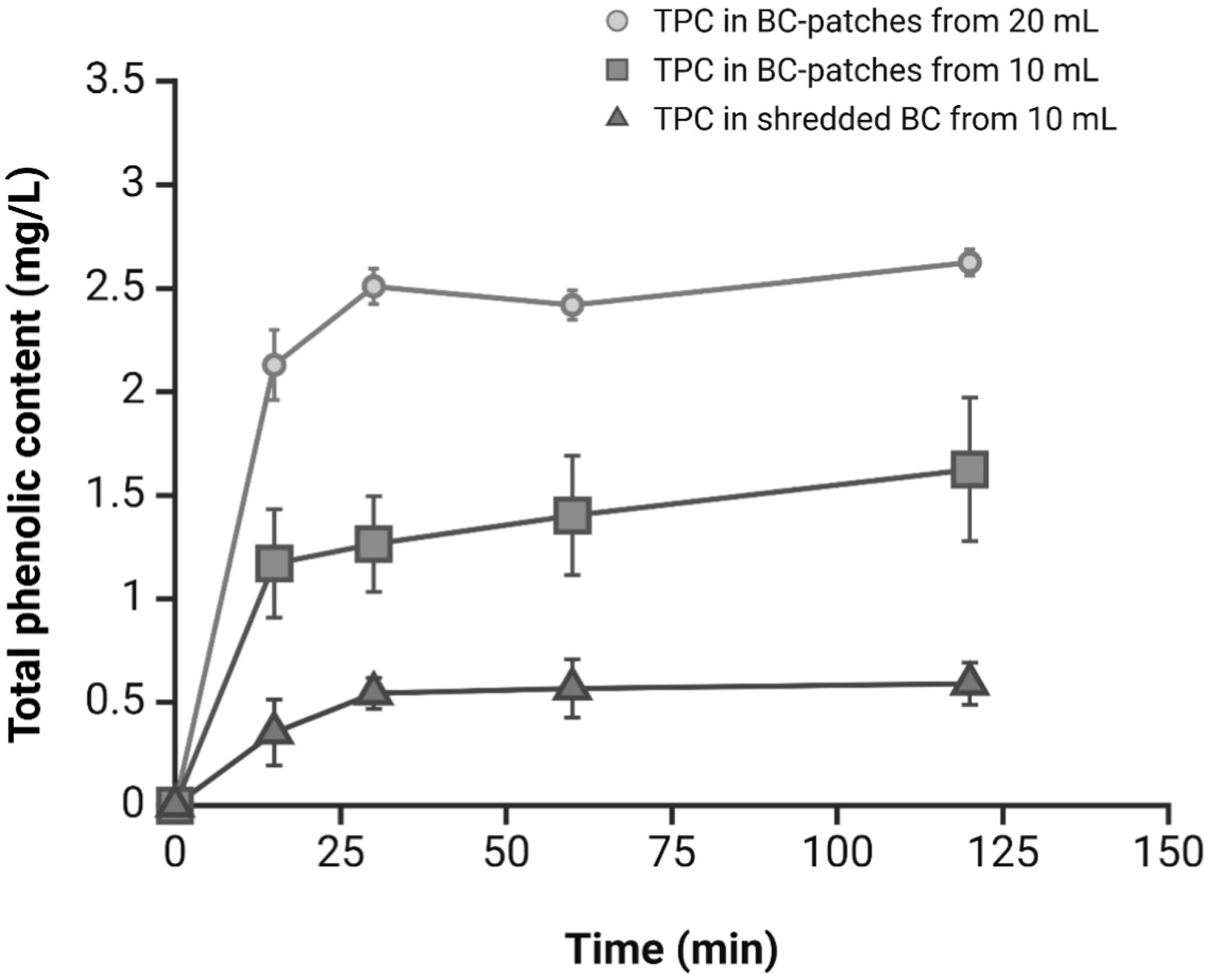
Total phenolic content (TPC) of different bacterial cellulose samples over time. The TPC is expressed in mg/L and measured in BC-patches incubated with 20 mL of APE (circle line), in BC-patches incubated with 10 mL of APE (square line) and in shredded-BC incubated with 10 mL of APE (triangle line). Data represent mean ± standard deviation. The extraction yield varied significantly depending on the BC format and volume, with BC patches from 20 mL showing the highest TPC levels, reaching a plateau around 50 min.

Incubation tests were conducted on patch-10, patch-20, and shredded-10 samples to assess the absorption capacity of BC. Before use, the cellulose samples exhibited a moisture content of 97% (w/w) and a thickness of approximately 2 mm. As shown in Figure 6, the cumulative absorption of polyphenols over 2 h incubation period for patches was in line with the literature,^14^ the absorption for shredded-BC, presented here for the first time, was lower than that of the patches. Furthermore, this absorption capacity increased with the initial polyphenol amount, rising from 10 to 20 mL of the APE. Upon closer analysis of the data, it is evident that polyphenol absorption exhibits a linear response for the patch group. Assuming the starting quantity of polyphenols obtained from the experiment with 250 g of apple waste, 46% of the polyphenols were absorbed from patch-10, while only 37% were absorbed from patch-20. Nevertheless, this discrepancy does not imply lower polyphenol content in patch-20; a comparison of the data in Figure 6 shows that the polyphenol content in patch-20 is approximately 38% higher than in patch-10. The absorption of shredded-10 is notably less effective than the patches, with only 17% of polyphenols being absorbed.

Data obtained from FT-IR analysis and reported in the following sections confirms that there are no molecular differences between the molecular chains that composed BC patches and those of shredded-BC. Thus, we can state that the interactions between cellulose and polyphenols are the same for both patches and shredded-BC. Consequently, the disparity in absorption can only be attributed to the different three-dimensional structures between the two forms. Polyphenol molecules absorbed on the surface exhibit fewer interactions than those embedded within the cellulose matrix, where they are completely surrounded by BC molecules. This allows for higher absorption in bulky BC-patches compared to shredded-BC.

Furthermore, data and kinetic trends indicate that shredded-10 reaches a plateau condition more rapidly, typically within 30 min. In comparison, the patches continue to absorb polyphenols at a slower rate beyond the initial 30 min, extending up to 120 min. Thus, we can state that the plateau is reached faster in the case of shredded-BC due to the more readily available interaction sites.

Based on the SEM results indicating that the size and percentage of pores in the two different cellulose matrices are comparable, the difference can be attributed to the greater surface area that shredded-BC possesses compared to the patches. From these results, it is evident that fragmented structures provide more readily available interaction sites. In contrast, patches have a smaller surface area, resulting in fewer immediately available sites. This observation suggests that the initial and rapid absorption of polyphenols within the first 30 min of incubation occurs primarily at the binding sites on the surface of the cellulose ribbons. Only after an extended period polyphenols penetrate the intricate cellulose matrix, accessing additional binding sites.

Although BC is suitable for the absorption of polyphenols, other carriers, such as pectin,^28^ have been reported to achieve higher polyphenol incorporation due to its ability to be extracted directly from fruit waste and its natural enrichment in polyphenols. However, a higher concentration does not necessarily translate into higher polyphenol release, considering the natural entrapment of polyphenols within the pectin matrix, and this release data is often not reported. A distinct advantage of BC is that postencapsulation allows for the selective enrichment of BC with specific polyphenols or other substances. This enables the production of more targeted final products for various applications, focusing on enhancing the effect of a few highly effective compounds.

### 3.4 Release Comparisons between BC-Patches and Shredded-BC

**Figure 7:**
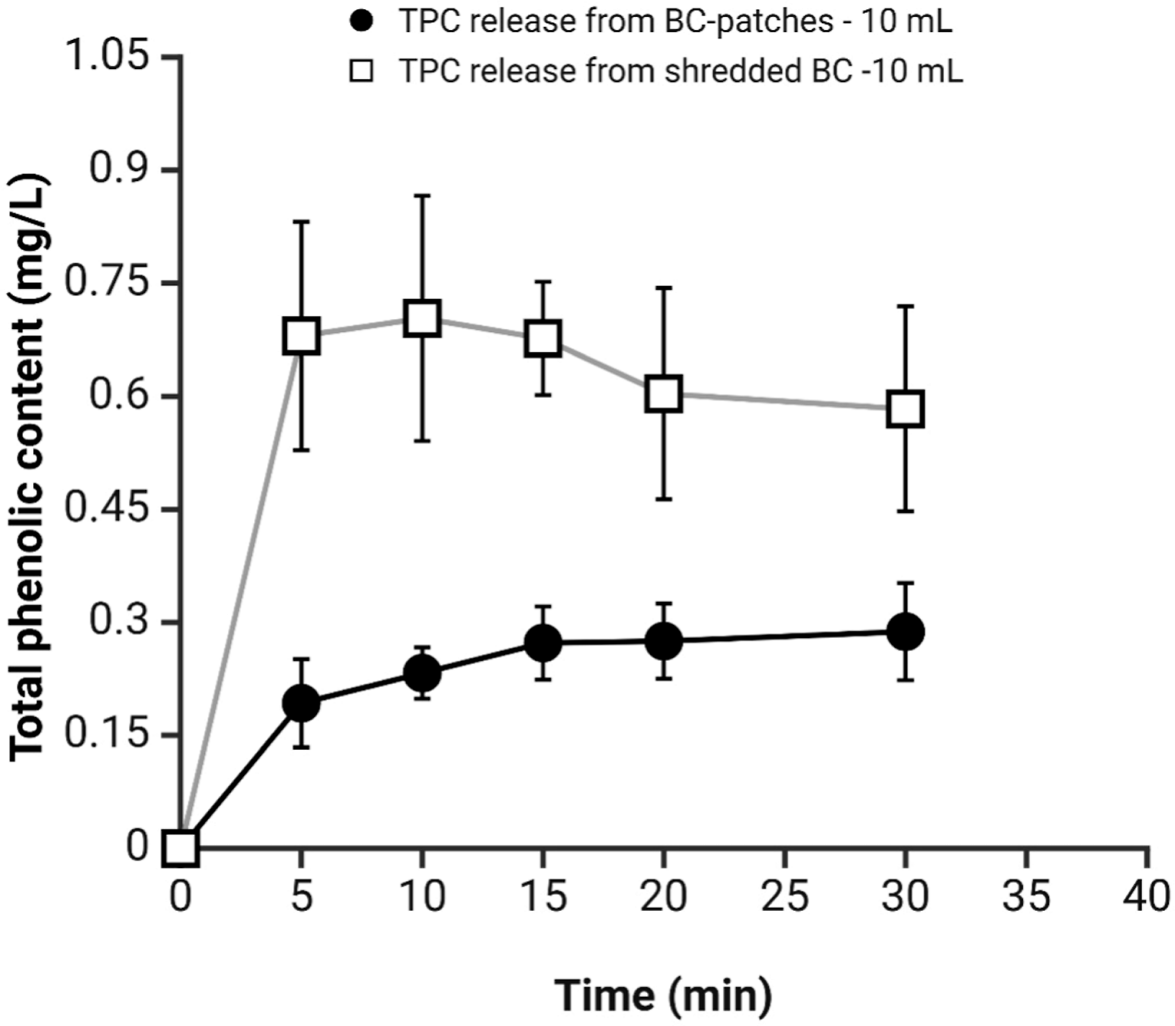
Release of total phenolic content from BC samples over time. The TPC is expressed in mg/L and measured for BC patches extracted from 10 mL (black circle line) and shredded-BC extracted from 10 mL (square line). Data represent mean ± standard deviation. Shredded-BC showed a faster and higher TPC release compared to BC patches, stabilizing after 10 min, while BC patches exhibited a gradual release over time.

To assess the release capacity of BC and ensure comparable results, we only focused on patch-10 and shredded-10. Samples of patch-10 and shredded-10 were immersed in 10 mL of PBS for 30 min, then, 500 μL samples collected every 5 min. The cumulative release content was measured, and the results are presented in Figure 7. In line with the results of the incubation tests, shredded-10 exhibited faster release kinetics compared to the patch. Notably, shredded-10 achieved the complete release of the absorbed polyphenols within just 5 min, whereas the patch required a longer duration, typically around 15 min. Once again, this difference in behavior can be attributed to the larger surface area of shredded-BC and less intricate matrix, which accelerate both the ingress and egress of polyphenols.

A notable distinction arises when comparing the amount of polyphenols released relative to the initial polyphenol load in the various systems under consideration. Shredded-BC demonstrates a near-complete release of approximately 98%, whereas patch-10 exhibits release percentages of 17%. This behavior can again be explained by the different arrangements of the cellulose fibers in the two systems.

After the initial 30 min of release, the PBS was replaced with a new 10 mL, and a release of approximately 0.14 mg/L GAE was evidenced during the next 30 min from patch-10. After this time, no significant release occurred for 2 h of incubation. Consequently, it is logical to assume that some of the absorbed polyphenols remained trapped within the patch.

The absorption and release kinetics findings of these three distinct systems underscore a clear trend: the higher the initial polyphenol content in the extract, the greater the absorption. Furthermore, while patches excel in uptake amount compared to shredded-BC, the latter excels in absorption and release rates.

Furthermore, the SEM results indicate that the rapid kinetics of shredded-BC are not a consequence of the material porosity, but of the surface area immediately available for absorption and release.

### 3.5 Fourier-Transform Infrared Spectroscopy Analysis

The intrinsic purity and crystalline attributes of BC are distinctive features. The subsequent confirmatory analysis of BC produced by SCOBY examined through FT-IR spectra indicated structural similarity and purity levels consistent with findings from a previous study reported in the literature.^8^ Additionally, FT-IR was employed to evaluate the influence of incorporating APE on the molecular forces between layers and components of BC.

As highlighted in Supporting Information Figures S1 and S2, the peaks at 3345.89 and 2917.77 cm^–1^ are attributed to the stretching valence vibration of free hydroxyl – OH and C–H stretching groups, respectively.^29^ Additionally, a peak at 1596.77 cm^–1^ indicated C=O stretching vibration. The bands observed at 1058.73 and 1033.66 cm^–1^ correspond to the elongation of the C–C single bonds, C–OH, and C–H ring. Moreover, in the fingerprint region, the stretching of the C–O–C bond at the β (1–4) glycosidic bond in cellulose, along with the O–H out-of-phase bending at wave numbers 669.18, 636.39, and 607.47 cm^–1^, were also detected.

**Figure 8:**
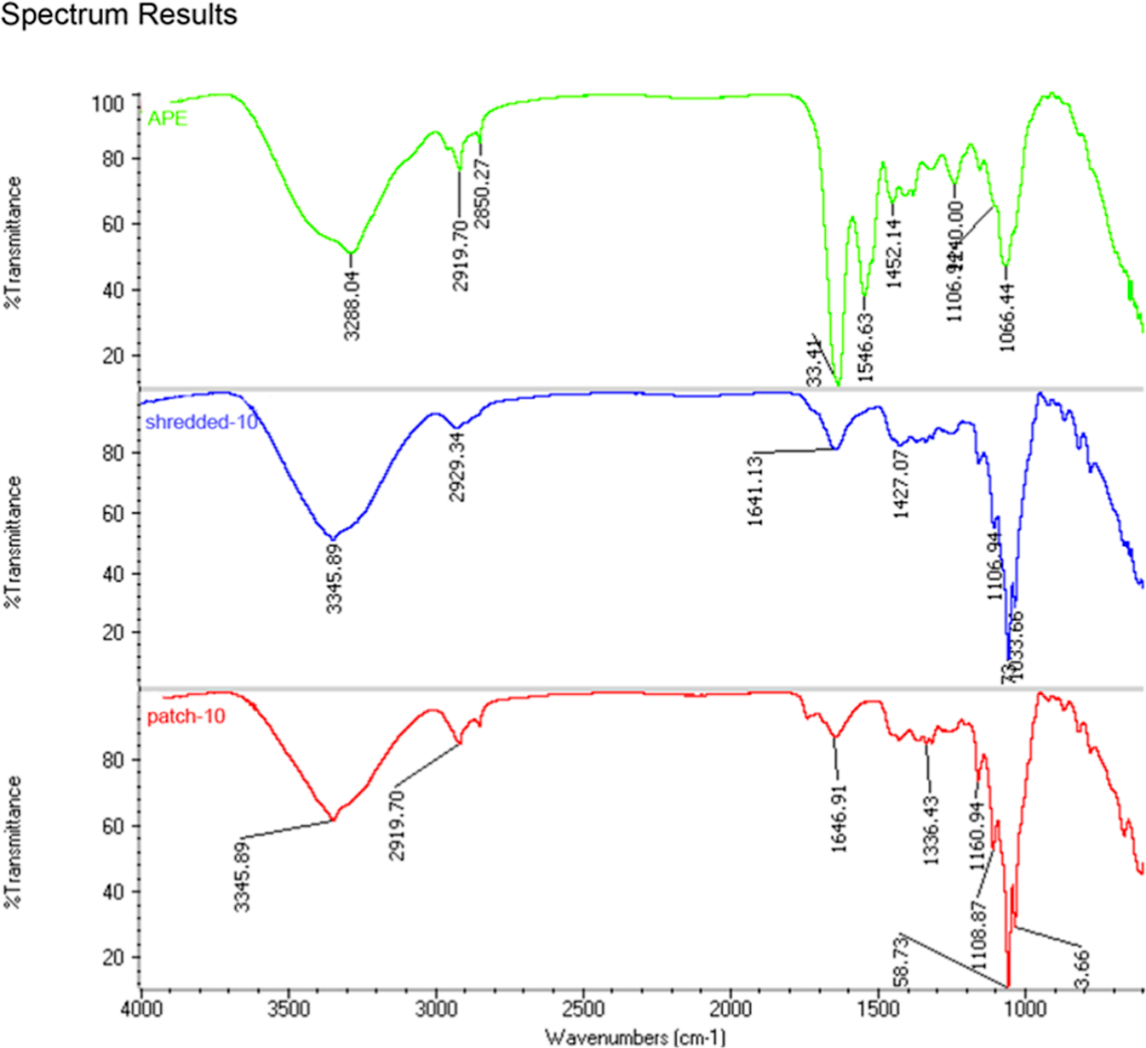
FT-IR spectra of apple polyphenol extract (APE, green) shredded bacterial cellulose (shredded-10, blue), and bacterial cellulose patch (patch-10, red). The spectra display characteristic peaks associated with functional groups, highlighting differences in chemical interactions among the samples. Key peaks include those corresponding to O–H stretching (∼3300 cm^–1^), C–H stretching (∼2919 cm^–1^), and C=O stretching (∼1641 cm^–1^). Variations in peak intensities and positions indicate differences in the composition and binding of polyphenols in the shredded and patch forms of bacterial cellulose.

A comparison was made of the FT-IR spectra of APE, shredded-10 and patch-10 in Figure 8. Noteworthy is the significant peak at 1633.41 cm^–1^ induced by the breathing vibration of benzene rings in apple extract. The presence of this peak is more substantial in APE (Supporting Information Figure S3). At the same time, it has a minor intensity in patch-10 (Supporting Information Figure S4) and Shredded-10 (Supporting Information Figure S5) depending on the absorption of polyphenols by BC.

When the characteristic peaks of layered and shredded-BC treated with APE were compared, it was found that no new characteristic peak arose when APE was introduced, indicating that no new covalent link was established between chemicals.^30^

The presence of APE should result in flattening the characteristic peaks associated with O-H stretching vibration between 3200 and 3500 cm^–1^, thus suggesting that APE, both in the layered and shredded-BC, formed hydrogen bonds, diminishing the stretching effect of accessible OH. Instead, analysis of the FT-IR spectra displays an increased peak intensity, which may be attributed to the fact that excess polyphenols increased the amount of –OH groups in the free state because they could not form more hydrogen bonds with the matrix.^31^

### 3.6 Biocompatibility Tests

**Figure 9:**
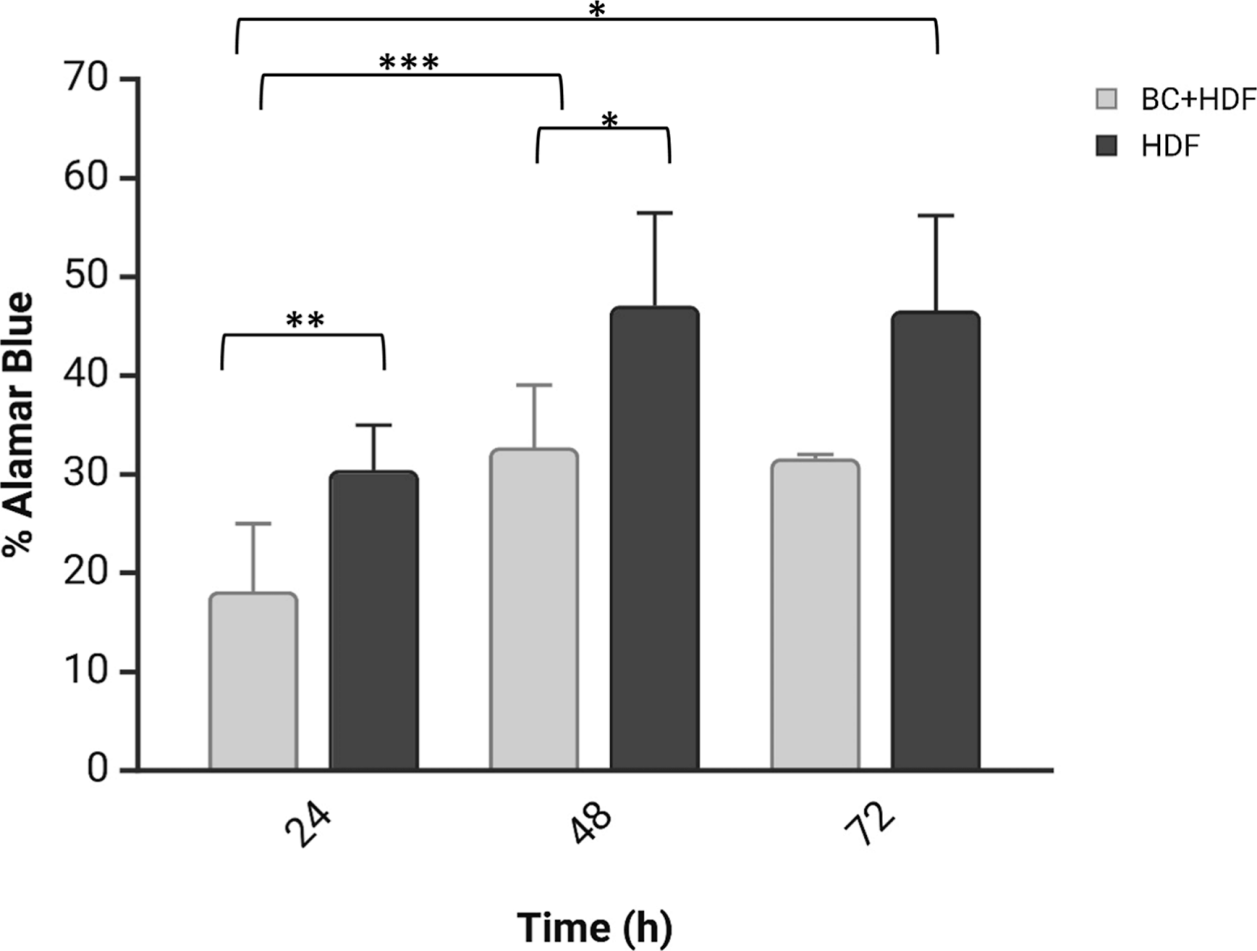
Cell viability of human dermal fibroblasts (HDF) cultured with bacterial cellulose (BC + HDF, light gray) and without bacterial cellulose (HDF, dark gray) over a 72 h experiment quantified using Alamar Blue assay. Data are expressed as mean ± standard deviation. Cell viability increased over time in both groups, with a slight enhancement observed in the presence of bacterial cellulose, quantified using Alamar Blue assay suggesting its potential biocompatibility and supportive role in cell growth. The asterisk * indicates a significant difference *p* < 0.05, **p < 0.01, ***p < 0.005.

The properties of biomaterials have long been recognized to affect cell behavior in terms of adhesion, viability and proliferation.^32^ The results of the Alamar Blue Assay for vitality testing on BC layer samples were obtained at 24, 48, and 72 h and subsequently compared with two-dimensional (2D) HDFs samples used as a control (Figure 9). The metabolic activity of cells causes the reduction in the Alamar Blue assay to change from an oxidized form (blue) to a fluorescent form (red), indicating cell viability. The percentage of reduction of HDFs adhered to the surface of the BC layer increased from 31.2 ± 7.7% at 24 h to 46.8 ± 0.6% at 72 h. This slight increase in the percentage reduction of HDFs on the BC layer from 24 to 72 h indicates that HDFs can proliferate on the BC surface.

Biocompatibility tests were performed on BC layer samples for convenience, as the continuous layer was more suitable for cell cultivation. Technically, shredded-BC retains the same biocompatibility as still BC but with a different shape.

## 4 Future Perspectives

In recent decades, bacterial cellulose has become increasingly used as a replacement material for classical cellulose extracted from plants, but above all as a carrier of substances. Due to its biocompatibility and rapid production by bacteria, it can currently be used in various fields such as food, paper, packaging, textiles and bioconcrete as well as bioremediation, cosmetics, electronics and sensing applications.

The proposed process flowchart may be easily scaled up for future industrial applications. Indeed, starting with the polyphenol extraction process, the RSLDE technique is already useable on a large scale. There are larger models of this extractor available than the one used in this study, and the yields are scalable by increasing the amounts of solid matrix and solvent used. Furthermore, the extraction process takes place at room temperature, so there is no need to consume energy to heat the system. Other advantages include the possibility of using green solvents and the reduced impact of matrix particle size on the final extract yield.^22^

The incubation process is also easily applicable on a large scale. If we instead consider the actual process of producing BC, SCOBY cultures are very fast in producing thin films, which can already be recovered after only 3 days. Subsequent autoclavation and bleaching are also methods that can be widely used on a large scale. Furthermore, thinking about shredded-BC production, there is no need to pay attention to possible rupture of BC layers, as they will have to be shredded afterward. Moreover, the production of shredded-BC includes only one simple extra step compared to the production of the layers, and since it is a purely mechanical step, the ease of achievement is optimal. Anyway, by optimizing the blending equipment in terms of time, power and amount of material to be processed, it is possible to obtain shredded-BC with the desired fragment size.

Returning to the possibility of adjusting fragment size, it can be thought of different release rates to apply to various biotechnological and cosmetic formulations, such as spreadable masks, scrubs, creams and emulsions. The possible addition of substances such as Vitamin C, propolis, resveratrol, anthocyanins, quercetin, epicatechin, which are easily absorbed to BC, can also enrich shredded-BC with regenerative, purifying, antiaging, antioxidant and antibacterial properties.

## 5 Conclusions

This study demonstrated the potential of bacterial cellulose (BC) in a shredded form as carrier for bioactive compounds and compared it with its traditional layered form. Shredding significantly influenced the polyphenol absorption and release kinetics of BC without altering its intrinsic microstructure. Initial experiments involved preparing a polyphenolic hydroalcoholic extract from industrial apple waste, which was then tested on both forms of BC.

The ability of cellulose to absorb polyphenols can be attributed to van der Waals forces and hydrogen bond interactions between the extract and the cellulosic matrix. These interactions occur due to the presence of hydroxyl groups in cellulose, which form bonds with the hydrogen and aromatic rings of polyphenols. As confirmed by SEM and FT-IR analyses, the intrinsic properties of porosity percentage, mean pore area, and molecular structure were not significantly different between the two BC forms. Thus, the observed differences in absorption and release kinetics are solely due to variations in their three-dimensional structures.

The increased surface-to-volume ratio and simplified structure of shredded-BC resulted in enhanced polyphenol absorption, with a faster plateau phase compared to the layered counterpart. Notably, shredded-BC exhibited a 2.5-fold faster absorption rate and achieved 98% release efficiency within 15 min, compared to only 17% for the layered form. These findings confirm that enhanced kinetics arise from structural differences rather than compositional changes, reinforcing the potential of shredded-BC as an innovative material for controlled-release applications by playing with BC particle size.

Beyond its ability to rapidly absorb and release bioactive compounds, shredded-BC offers a more predictable release profile, making it a promising candidate for cosmetic formulations such as spreadable masks and other controlled-delivery systems. Its biocompatibility, combined with improved functionality, suggests broader applications in the biotechnological and pharmaceutical industries. Future studies should explore its interactions with other bioactive molecules, assess its stability over time, and investigate its in vivo effects to expand its potential in skincare, drug delivery, and regenerative medicine.

## Data Availability

The data sets generated during and analyzed during the current study are available from the corresponding authors upon reasonable request.

## Supporting Information Available

Revised—Supporting Information_Experimental results of FT-IR spectra. FT-IR spectra of BC-patch, shredded-BC, APE, BC-patch and shredded-BC incubated with APE (PDF)

## References

[ref1] Gregory D. A.; Tripathi L.; Fricker A. T. R.; Asare E.; Orlando I.; Raghavendran V.; Roy I. Bacterial Cellulose: A Smart Biomaterial with Diverse Applications. Mater. Sci. Eng. R Rep. 2021, 145, 100623 10.1016/j.mser.2021.100623.

[ref2] Klemm D.; Schumann D.; Udhardt U.; Marsch S. Bacterial Synthesized Cellulose D̵ Artificial Blood Vessels for Microsurgery. Prog. Polym. Sci. 2001, 26, 1561–1603. 10.1016/s0079-6700(01)00021-1.

[ref3] Mbituyimana B.; Liu L.; Ye W.; Ode Boni B. O.; Zhang K.; Chen J.; Thomas S.; Vasilievich R. V.; Shi Z.; Yang G. Bacterial Cellulose-Based Composites for Biomedical and Cosmetic Applications: Research Progress and Existing Products. Carbohydr. Polym. 2021, 273, 118565 10.1016/j.carbpol.2021.118565. 34560976

[ref4] Nilforoushzadeh M. A.; Amirkhani M. A.; Zarrintaj P.; Moghaddam A. S.; Mehrabi T.; Alavi S.; Mollapour Sisakht M. Skin Care and Rejuvenation by Cosmeceutical Facial Mask. J. Cosmet. Dermatol. 2018, 17 (5), 693–702. 10.1111/jocd.12730. 30133135

[ref5] Perugini P.; Bleve M.; Redondi R.; Cortinovis F.; Colpani A. In Vivo Evaluation of the Effectiveness of Biocellulose Facial Masks as Active Delivery Systems to Skin. J. Cosmet. Dermatol. 2020, 19 (3), 725–735. 10.1111/jocd.13051. 31301106 PMC7027794

[ref6] Silva N. H. C. S.; Drumond I.; Almeida I. F.; Costa P.; Rosado C. F.; Neto C. P.; Freire C. S. R.; Silvestre A. J. D. Topical Caffeine Delivery Using Biocellulose Membranes: A Potential Innovative System for Cellulite Treatment. Cellulose 2014, 21 (1), 665–674. 10.1007/s10570-013-0114-1.

[ref7] Pacheco G.; De Mello C. V.; Chiari-Andréo B. G.; Isaac V. L. B.; Ribeiro S. J. L.; Pecoraro E. ´.; Trovatti E. Bacterial Cellulose Skin Masks-Properties and Sensory Tests. J. Cosmet. Dermatol. 2018, 17 (5), 840–847. 10.1111/jocd.12441. 28963772

[ref8] Di Natale C.; De Gregorio V.; Lagreca E.; Mauro F.; Corrado B.; Vecchione R.; Netti P. A. Engineered Bacterial Cellulose Nanostructured Matrix for Incubation and Release of Drug-Loaded Oil in Water Nanoemulsion. Front. Bioeng. Biotechnol. 2022, 10, 851893 10.3389/fbioe.2022.851893. 35356776 PMC8959586

[ref9] Fernandes I. D. A. A.; Maciel G. M.; Ribeiro V. R.; Rossetto R.; Pedro A. C.; Haminiuk C. W. I. The Role of Bacterial Cellulose Loaded with Plant Phenolics in Prevention of UV-Induced Skin Damage. Carbohydr. Polym. Technol. Appl. 2021, 2, 100122 10.1016/j.carpta.2021.100122.

[ref10] Abbas M.; Saeed F.; Anjum F. M.; Afzaal M.; Tufail T.; Bashir M. S.; Ishtiaq A.; Hussain S.; Suleria H. A. R. Natural Polyphenols: An Overview. Int. J. Food Prop. 2017, 20 (8), 1689–1699. 10.1080/10942912.2016.1220393.

[ref11] Zillich O. V.; Schweiggert-Weisz U.; Eisner P.; Kerscher M. Polyphenols as Active Ingredients for Cosmetic Products. Int. J. Cosmet. Sci. 2015, 37 (5), 455–464. 10.1111/ics.12218. 25712493

[ref12] Bordenave N.; Hamaker B. R.; Ferruzzi M. G. Nature and Consequences of Non-Covalent Interactions between Flavonoids and Macronutrients in Foods. Food Funct. 2014, 5 (1), 18–34. 10.1039/C3FO60263J. 24326533

[ref13] Phan A. D. T.; Netzel G.; Wang D.; Flanagan B. M.; D’Arcy B. R.; Gidley M. J. Binding of Dietary Polyphenols to Cellulose: Structural and Nutritional Aspects. Food Chem. 2015, 171, 388–396. 10.1016/j.foodchem.2014.08.118. 25308685

[ref14] Liu D.; Martinez-Sanz M.; Lopez-Sanchez P.; Gilbert E. P.; Gidley M. J. Adsorption Behaviour of Polyphenols on Cellulose Is Affected by Processing History. Food Hydrocolloids 2017, 63, 496–507. 10.1016/j.foodhyd.2016.09.012.

[ref15] Nowak A.; Ossowicz-Rupniewska P.; Rakoczy R.; Konopacki M.; Perużyńska M.; Droździk M.; Makuch E.; Duchnik W.; Kucharski Ł.; Wenelska K.; Klimowicz A. Bacterial Cellulose Membrane Containing Epilobium Angustifolium L. Extract as a Promising Material for the Topical Delivery of Antioxidants to the Skin. Indian J. Manag. Sci. 2021, 22 (12), 6269 10.3390/ijms22126269. PMC8230535 34200927

[ref16] Fatima A.; Yasir S.; Khan M. S.; Manan S.; Ullah M. W.; Ul-Islam M. Plant Extract-Loaded Bacterial Cellulose Composite Membrane for Potential Biomedical Applications. J. Bioresour. Bioprod. 2021, 6 (1), 26–32. 10.1016/j.jobab.2020.11.002.

[ref17] Indrianingsih A. W.; Rosyida V. T.; Apriyana W.; Hayati S. N.; Darsih C.; Nisa K.; Ratih D. Antioxidant and Antibacterial Properties of Bacterial Cellulose— Indonesian Plant Extract Composites for Mask Sheet. J. Appl. Pharm. Sci. 2020, 10, 037 10.7324/JAPS.2020.10705.

[ref18] Bandyopadhyay S.; Saha N.; Saha P. Characterization of Bacterial Cellulose Produced Using Media Containing Waste Apple Juice. Appl. Biochem. Microbiol. 2018, 54 (6), 649–657. 10.1134/S0003683818060042.

[ref19] Sommer A.; Staroszczyk H. Bacterial Cellulose vs. Bacterial Cellulose Nanocrystals as Stabilizer Agents for O/W Pickering Emulsions. Food Hydrocolloids 2023, 145, 109080 10.1016/j.foodhyd.2023.109080.

[ref20] Martins D.; Rocha C.; Dourado F.; Gama M. Bacterial Cellulose-Carboxymethyl Cellulose (BC:CMC) Dry Formulation as Stabilizer and Texturizing Agent for Surfactant-Free Cosmetic Formulations. Colloids Surf., A 2021, 617, 126380 10.1016/j.colsurfa.2021.126380.

[ref21] Posadino A.; Biosa G.; Zayed H.; Abou-Saleh H.; Cossu A.; Nasrallah G.; Giordo R.; Pagnozzi D.; Porcu M.; Pretti L.; Pintus G. Protective Effect of Cyclically Pressurized Solid–Liquid Extraction Polyphenols from Cagnulari Grape Pomace on Oxidative Endothelial Cell Death. Molecules 2018, 23 (9), 2105 10.3390/molecules23092105. 30134642 PMC6225102

[ref22] Naviglio D.; Scarano P.; Ciaravolo M.; Gallo M. Rapid Solid-Liquid Dynamic Extraction (RSLDE): A Powerful and Greener Alternative to the Latest Solid-Liquid Extraction Techniques. Foods 2019, 8 (7), 245 10.3390/foods8070245. 31284507 PMC6678328

[ref23] Naviglio D.; Trifuoggi M.; Varchetta F.; Nebbioso V.; Perrone A.; Avolio L.; De Martino E.; Montesano D.; Gallo M. Efficiency of Recovery of the Bioactive Principles of Plants by Comparison between Solid–Liquid Extraction in Mixture and Single-Vegetable Matrices via Maceration and RSLDE. Plants 2023, 12 (16), 2900 10.3390/plants12162900. 37631112 PMC10458922

[ref24] Kupina S.; Fields C.; Roman M. C.; Brunelle S. L. Determination of Total Phenolic Content Using the Folin-C Assay: Single-Laboratory Validation, First Action 2017.13. J. AOAC Int. 2018, 101 (5), 1466–1472. 10.5740/jaoacint.18-0031. 29895350

[ref25] Szerlauth A.; Muráth S.; Viski S.; Szilagyi I. Radical Scavenging Activity of Plant Extracts from Improved Processing. Heliyon 2019, 5 (11), e02763 10.1016/j.heliyon.2019.e02763. 31844703 PMC6895678

[ref26] Mauro F.; Corrado B.; De Gregorio V.; Lagreca E.; Di Natale C.; Vecchione R.; Netti P. A. Exploring the Evolution of Bacterial Cellulose Precursors and Their Potential Use as Cellulose-Based Building Blocks. Sci. Rep. 2024, 14 (1), 11613 10.1038/s41598-024-62462-9. 38773229 PMC11109180

[ref27] Vrhovsek U.; Rigo A.; Tonon D.; Mattivi F. Quantitation of Polyphenols in Different Apple Varieties. J. Agric. Food Chem. 2004, 52 (21), 6532–6538. 10.1021/jf049317z. 15479019

[ref28] Podetti C.; Riveros-Gomez M.; Román M. C.; Zalazar-García D.; Fabani M. P.; Mazza G.; Rodríguez R. Polyphenol-Enriched Pectin from Pomegranate Peel: Multi-Objective Optimization of the Eco-Friendly Extraction Process. Molecules 2023, 28 (22), 7656 10.3390/molecules28227656. 38005378 PMC10675440

[ref29] Hamed D. A.; Maghrawy H. H.; Abdel Kareem H. Biosynthesis of Bacterial Cellulose Nanofibrils in Black Tea Media by a Symbiotic Culture of Bacteria and Yeast Isolated from Commercial Kombucha Beverage. World J. Microbiol. Biotechnol. 2023, 39 (2), 48 10.1007/s11274-022-03485-0. PMC9768004 36538179

[ref30] Lin L.; Peng S.; Shi C.; Li C.; Hua Z.; Cui H. Preparation and Characterization of Cassava Starch/Sodium Carboxymethyl Cellulose Edible Film Incorporating Apple Polyphenols. Int. J. Biol. Macromol. 2022, 212, 155–164. 10.1016/j.ijbiomac.2022.05.121. 35609834

[ref31] Li X.; Liu Y.; Luo B.; Xiang W.; Chen Z. Effect of Apple Polyphenols on Physicochemical Properties of Pea Starch/Pulp Cellulose Nanofiber Composite Biodegradable Films. Int. J. Biol. Macromol. 2024, 257, 128480 10.1016/j.ijbiomac.2023.128480. 38052284

[ref32] Xi Loh E. Y.; Fauzi M. B.; Ng M. H.; Ng P. Y.; Ng S. F.; Ariffin H.; Mohd Amin M. C. I. Cellular and Molecular Interaction of Human Dermal Fibroblasts with Bacterial Nanocellulose Composite Hydrogel for Tissue Regeneration. ACS Appl. Mater. Interfaces 2018, 10 (46), 39532–39543. 10.1021/acsami.8b16645. 30372014

