# Supplementary material for: Shredded Bacterial Cellulose as a Potential Carrier of Polyphenols Derived from Apple Waste Applying Rapid Solid–Liquid Dynamic Extraction
Source: ACS Omega. 2025 Apr 3;10(14):14010–9. doi: 10.1021/acsomega.4c10689 (PMC12004162; doi:10.1021/acsomega.4c10689)

Supporting information

Shredded Bacterial Cellulose as a Potential Carrier of Polyphenols derived from Apple Waste applying Rapid Solid-Liquid Dynamic Extraction

Roberta Baldassini [1†], Brunella Corrado[2†], Elena Lagreca[3], Raffaele Vecchione[3*], Alma Sardo[5], Daniele Naviglio[6] and Paolo Antonio Netti[2-3-4]

[1] Knowledge for Business, via Manzoni 110, 80123, Naples, Italy

[2] Interdisciplinary Research Centre on Biomaterials (CRIB), University of Naples Federico II, Piazzale Tecchio 80, 80125, Naples, Italy

[3] Italian Institute of Technology, Largo Barsanti e Matteucci 53, 80125, Naples, Italy

[4] Department of Chemical, Materials and Industrial Production Engineering, University of Naples Federico II, Piazzale Tecchio 80, 80125, Naples, Italy

[5] Department of Veterinary Medicine and Animal Production, University of Naples Federico II, Via Federico Delpino, 80137, Naples, Italy

[6] Department of Chemical Science, University of Naples Federico II, Via Cintia 4, 80126, Naples, Italy

† These authors contributed equally

* email: raffaele.vecchione@iit.it

Find peaks results

Peaks and Height from BC patch FT-IR specrta **(Figure S1)**

Region	Peak	Height
1	644.11	87.38
1	667.25	85.39
1	1033.66	79.60
1	1056.80	76.15
1	1108.87	86.17
1	1162.87	91.98
1	1317.14	95.12
1	1427.07	96.23
1	1641.13	97.11
1	2917.77	98.29
1	3345.89	91.77

Figure S1 - BC patch FT-IR spectra

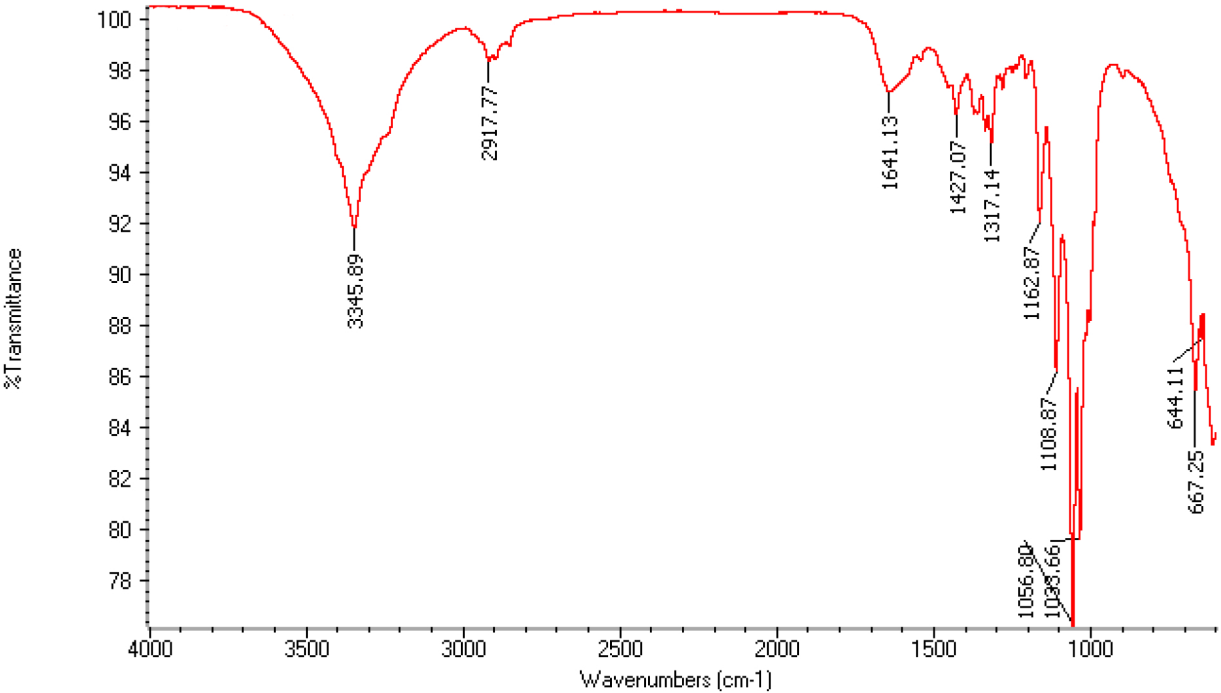

Find peaks results

Peaks and Height from BC shredded FT-IR spectra **(Figure S2)**

Region	Peak	Height
1	607.47	90.17
1	636.39	91.86
1	669.18	91.79
1	1033.66	90.06
1	1058.73	87.99
1	1108.87	92.95
1	1160.94	96.10
1	1315.21	97.55
1	1427.07	95.94
1	1596.77	97.89
1	2917.77	99.21
1	3345.89	96.36

Figure S2 - BC shredded FT-IR spectra

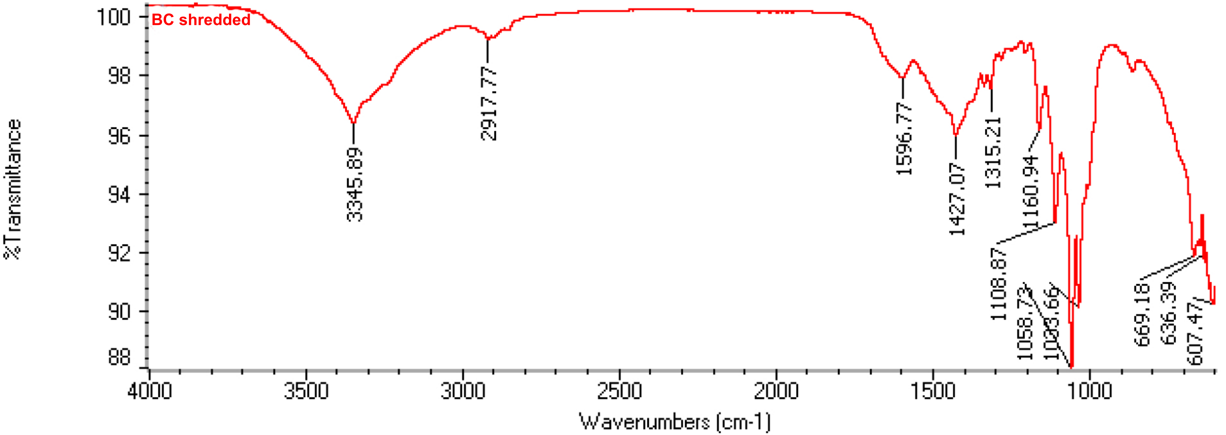

Find peaks results

Peaks and heights from APE FT-IR spectra **(Figure S3)**

Region	Peak	Height
1	644.11	82.89
1	1064.51	88.07
1	1240.00	93.39
1	1452.14	93.27
1	1546.63	88.57
1	1633.41	83.94
1	2919.70	95.53
1	3288.04	91.03

Figure S3 - APE FT-IR spectra

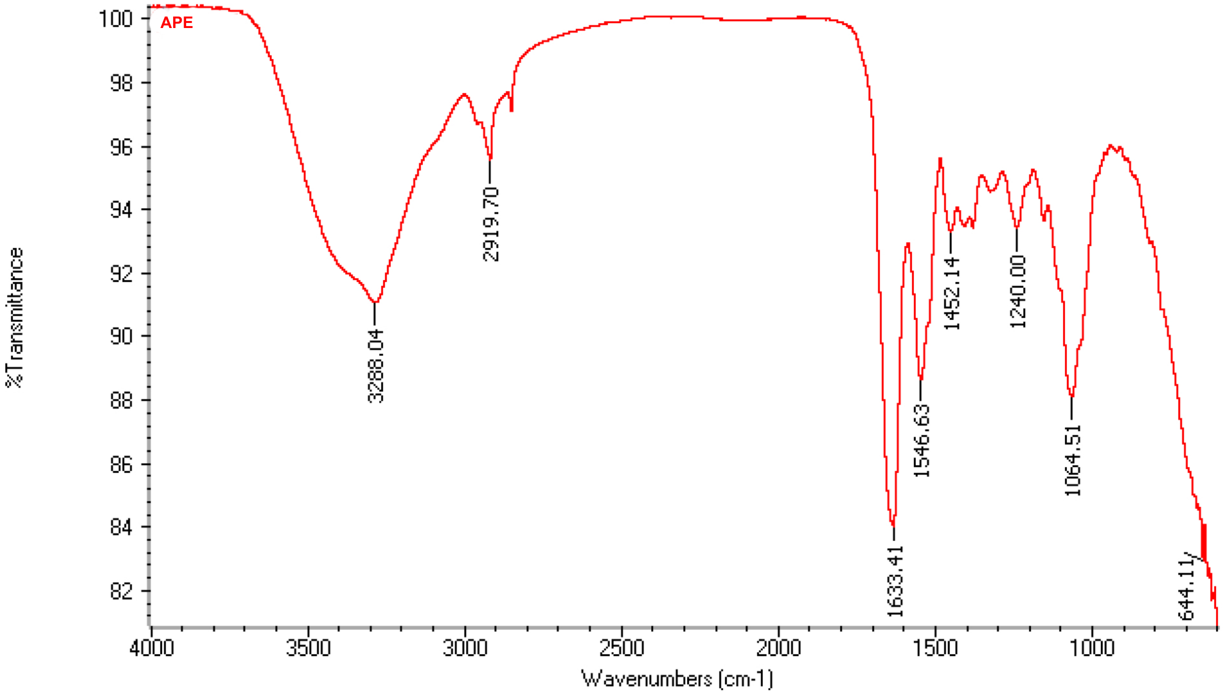

Find peaks results

Peaks and Height from patch-10 FT-IR spectra **(Figure S4)**

Region	Peak	Height
1	663.39	81.09
1	1033.66	76.19
1	1058.73	71.10
1	1108.87	83.47
1	1160.94	89.61
1	1317.14	93.71
1	1644.98	95.49
1	2919.70	94.95
1	3345.89	88.11

Figure S4 - Patch-10 FT-IR spectra

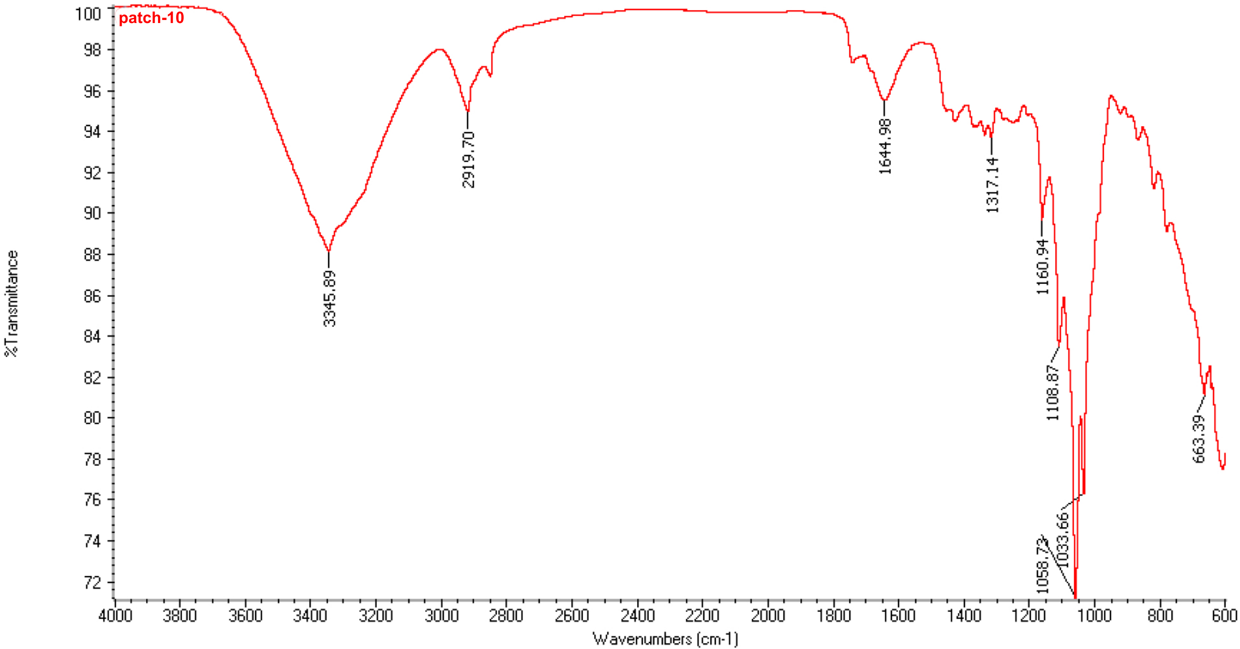

Find peaks results

Peaks and Height from shredded-10 FT-IR specrta **(Figure S5)**

Region	Peak	Height
1	817.67	89.31
1	1033.66	77.67
1	1058.73	73.32
1	1106.94	84.93
1	1336.43	93.64
1	1641.13	94.21
1	2929.34	96.19
1	3345.89	86.26

Figure S5 - Shredded-10 FT-IR spectra

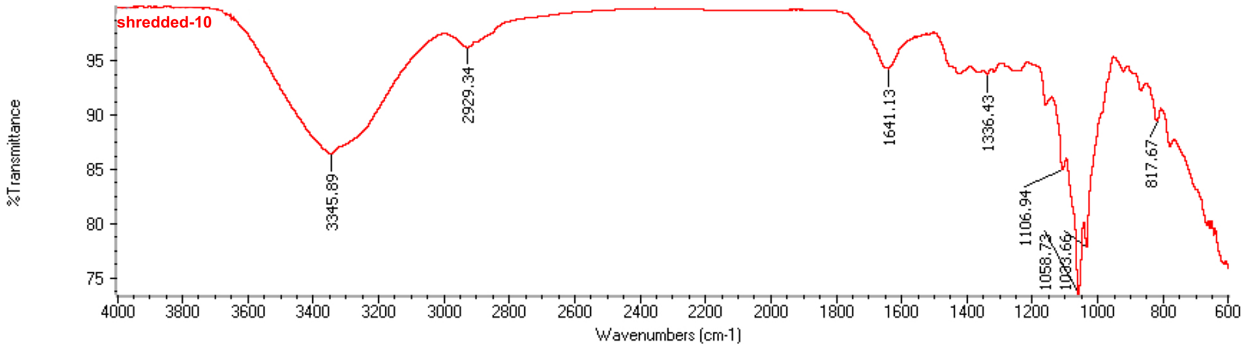

Supplement: Supplementary file 1 — ao4c10689_si_001.pdf [file ao4c10689_si_001.pdf]
